# Sodium Toxicity in the Nutritional Epidemiology and Nutritional Immunology of COVID-19

**DOI:** 10.3390/medicina57080739

**Published:** 2021-07-22

**Authors:** Ronald B. Brown

**Affiliations:** School of Public Health Sciences, University of Waterloo, Waterloo, ON N2L 3G1, Canada; r26brown@uwaterloo.ca

**Keywords:** COVID-19, coronavirus, SARS-CoV-2, sodium toxicity, nutritional epidemiology, nutritional immunology, virology, pathophysiology, mucosal immune system, pulmonary edema

## Abstract

Dietary factors in the etiology of COVID-19 are understudied. High dietary sodium intake leading to sodium toxicity is associated with comorbid conditions of COVID-19 such as hypertension, kidney disease, stroke, pneumonia, obesity, diabetes, hepatic disease, cardiac arrhythmias, thrombosis, migraine, tinnitus, Bell’s palsy, multiple sclerosis, systemic sclerosis, and polycystic ovary syndrome. This article synthesizes evidence from epidemiology, pathophysiology, immunology, and virology literature linking sodium toxicological mechanisms to COVID-19 and SARS-CoV-2 infection. Sodium toxicity is a modifiable disease determinant that impairs the mucociliary clearance of virion aggregates in nasal sinuses of the mucosal immune system, which may lead to SARS-CoV-2 infection and viral sepsis. In addition, sodium toxicity causes pulmonary edema associated with severe acute respiratory syndrome, as well as inflammatory immune responses and other symptoms of COVID-19 such as fever and nasal sinus congestion. Consequently, sodium toxicity potentially mediates the association of COVID-19 pathophysiology with SARS-CoV-2 infection. Sodium dietary intake also increases in the winter, when sodium losses through sweating are reduced, correlating with influenza-like illness outbreaks. Increased SARS-CoV-2 infections in lower socioeconomic classes and among people in government institutions are linked to the consumption of foods highly processed with sodium. Interventions to reduce COVID-19 morbidity and mortality through reduced-sodium diets should be explored further.

## 1. Introduction

Since coronavirus disease-2019 (COVID-19) was designated a pandemic by the World Health Organization on 11 March 2020 [1], there has been strong public demand for vaccines and pharmacotherapies to treat the disease [2], while modifiable dietary and nutritional factors for COVID-19 prevention remain relatively under-investigated. An urgent need has been identified for the research of non-drug interventions in COVID-19, including interventions for the modification of environmental disease risk factors [3]. As the potential for new outbreaks pose an imminent threat, the need for novel interventions is especially urgent for older adults in the high-risk category for morbidity and mortality from COVID-19 [4].

Still, a causal relationship may seem unlikely between dietary factors and COVID-19 etiology, an infectious influenza-like illness (ILI) [5] associated with severe acute respiratory syndrome coronavirus 2 (SARS-CoV-2). However, until the last few decades of the 20th century, causal relationships also seemed unlikely for dietary factors in non-communicable diseases like cancer and cardiovascular disease, the leading causes of death globally [6], but diet and nutrition research has since emerged in these areas and accelerated to the present [7]. Similarly, emerging evidence suggests that sodium toxicity, the toxic effect in the body caused by dysregulated amounts of the essential dietary micronutrient sodium [8,9], has potential causal influences in the etiology of ILIs like COVID-19. 

A high sodium intake is a dietary risk factor associated with multiple diseases, and it is estimated to have caused a mean of 3 million deaths globally in 2017 [10]. Several of these diseases have also been identified as underlying conditions associated with increased risk for COVID-19 morbidity and mortality, implying a causal link to sodium toxicity through transitive inference. Recently, nutritional status has been proposed to provide potential immunomodulary and anti-inflammatory benefits in COVID-19 and its comorbidities [11]. Nutritional immunology is a new discipline that studies the interplay between food and food components with immune responses and disease prevention [12]. The World Health Organization listed immunocompromised status and nutritional status as factors that increase susceptibility to infection [13]. The current need to identify the toxicological mechanisms associated with COVID-19 also remains urgent [14]. 

This article reviews a wide variety of research findings with the aim of synthesizing evidence of sodium toxicity as a modifiable dietary factor associated with the nutritional epidemiology and nutritional immunology of COVID-19 and SARS-CoV-2 infection. Grounded theory was used as a method to review the literature, which added scholarly rigor and objectivity when researching and writing this perspective paper [15]. The selected data consisted of research findings and evidence-based concepts from relevant knowledge domains, including epidemiology, virology, immunology, and clinical pathophysiology. Data were searched for using Google, Google Scholar, PubMed, and Scopus, as well as other books, journals, and databases available through the University of Waterloo Library. Starting with a clean slate to remove subjective opinions and assumptions, rigorous comparative analysis was used to scrutinize the literature and logically connect evidence-based findings into causative, associative, and mediating relationships. A more detailed description of this method is explained in a source written by the present author [16]. As categorical themes inductively formed through iterative analysis, theoretical saturation was reached in this paper (when no more new knowledge was obtained). The final result is a coherent and novel explanatory grounded theory linking sodium toxicity to the etiology of COVID-19 and SARS-CoV-2 infection. The paper proceeds with an overview of viral infection.

## 2. Current Perspective of Viral Infection

The basic structure of a virion consists of a piece of nucleic acid surrounded by a protein capsid [17]. Currently, the human virome is the most recently studied part of the human microbiome, but knowledge and research technology in this area are limited [18]. Distinctive viromes have been identified in the gastrointestinal tract, salivary glands, and respiratory tract. However, it is unclear which viruses have detrimental or beneficial effects on human health, and the molecular and physiological mechanisms of these effects are unknown. Whether a virus is harmful or harmless can depend on the immunological status and health of the host [19]. 

An analysis of the transcriptomic architecture of SARS-CoV-2, which analyzes how the genes of the coronavirus are translated from a single strand of ribonucleic acid (RNA), found that the shortened tails of the virus might represent aged and decay-prone RNA (as in disposable genetic waste products), similar to the type of RNA generated by the mitochondria [20]. Mitochondria organelles in cells contain their own disposable mitochondrial deoxyribonucleic acid (mtDNA), which is densely packed in nucleoids, separate from the genes in the cell nucleus [21]. The tight density of mtDNA packing is similar to the density of DNA tightly packed into the capsid of a papillomavirion [22]. The turnover of mtDNA has a relatively brief half-life, and degraded mtDNA may be abandoned without noticeable physiological harm to the mitochondria. Mammalian somatic cells can contain up to 2000 mitochondria per cell [23], with each mitochondria potentially contributing genetic waste products. Additionally, bacteria in the microbiome contain chromosomal DNA packed in a nucleoid [24], which can also contribute to genetic waste when the cell expires. 

Cells have waste removal and recycling mechanisms that manage genetic waste products [25]. Proteins labelled with ubiquitin are targeted and broken down by proteasomes for reuse in synthesizing new proteins. Lysosomes contain digestive enzymes to breakdown organelles and viruses during the process of autophagy, and collected piles of unwanted cellular trash form aggregates, which might explain the formation of viral aggregates [26]. Extracellular vesicles (EVs) are also formed, and cargo is loaded and released by cells to function as a waste management mechanism that removes intracellular debris [27]. In pathological conditions, EVs may not clear promptly from circulation, contributing to the underlying pathology. 

Some EVs may resemble the structure and function of viruses, carrying viral proteins and genome fragments that make these EVs indistinguishable from “noninfectious” or “defective” viruses, which do not fit the prevailing definition of infectious viral agents that multiply exclusively in living cells [28]. Genome fragments in EVs include discarded messenger RNA (mRNA), a genetic messenger molecule [29]. Importantly, both nuclear and mitochondria mRNA undergo degradation after having been translated during protein synthesis [30,31], and very small EVs called exosomes have been found to transport mostly mRNA fragments [32]. 

Evidence of the role played by noninfectious viruses in pathogenesis is considered “very limited” and “obscure” by textbook authors writing on virus replication [33]. However, the biological process of natural infection is much more complex than the oversimplified depiction in textbooks. For example, even though noninfectious viruses make up most of the viral population in influenza A infection, the range of biological activity attributed to noninfectious viruses, including self-aggregation, has been understudied and “does not necessarily imply a defect of any kind” [34]. Furthermore, nucleic acid detection of high viral loads in clinical specimens collected during a pandemic cannot distinguish between noninfectious viruses and viruses that are assumed to be proliferating, nor is detection of viral RNA in specimens always correlated with viral transmissibility [35].

A recent discovery found that complete genomic mtDNA can be harbored in EVs [36], providing further evidence of a transport mechanism in which genomic debris is circulated for waste clearance, and which may contribute to the pathology if the waste is allowed to accumulate as an aggregate. Collective viral spread by virion aggregates helps a viral infection overcome infection barriers [37], which raises the possibility that the observed association of multiplication with viral infection could be caused by viral aggregation from accumulated waste products. For example, in a viral replication experiment, viruses added to cultured cells disappeared and reappeared later as progeny [33]. However there are potential flaws in this “proof of concept” experiment. Virion particle breakdown and recycling by the cultured cells’ immune response could explain the “remarkable” disappearance of the viruses, and gradual accumulation of the cultured cells’ own genetic waste products, i.e., exosomes containing genome fragments, could explain the putative reappearance of a viral progeny. Furthermore, no comparisons with the results from control groups were mentioned in this “experiment.” 

An infectious virus is claimed to hijack a host cell’s DNA reproductive mechanisms in order to translate the virus genome and replicate the virus [38]. However, the genetic code in the mRNA, which is a transcribed message destined for delivery to the ribosomes, can only be used to translate the biogenesis of harmless proteins needed by the cell. Translation of the coded genetic message in mRNA fragments does not replicate the mRNA itself, making the fragments noninfectious. Similarly, RNA in SARS-CoV-2 contains 29,811 nucleotides that encode 29 proteins, most of which are unrelated to the structure of mRNA itself [39], implying that the virus, composed of noninfectious mRNA fragments, is itself noninfectious. If true, this further implies that SARS-CoV-2 cannot replicate within a host. The presence of noninfectious viruses could also explain why viral RNA is detected in a host even if a viral infection is not present [40]. In addition, mutations claimed to occur in influenza viruses are reassortments of RNA fragments, often packaged in groups of eight fragments, and more knowledge of the mechanisms in genome packaging is needed [41]. Furthermore, single virions are rarely sufficient to establish infections, and the dispersion of multi-virion structures in infection remain poorly understood, needing more study in this area of virology [42].

EVs, including apoptotic bodies, exosomes, and microvesicles, are also responsible for the transmission of biomolecules in the development of sepsis [43]. Viral sepsis has been proposed as a critical immunopathological mechanism in COVID-19, based on findings of significantly elevated levels of cytokines, chemokines, and other immunological response agents in COVID-19 cases—part of a cytokine storm often seen in severe influenza infections [44]. Sepsis from infection is a complicated syndrome of pathophysiological mechanisms that are not yet fully understood. A little less than half of sepsis cases have non-bacterial causes, and viral sepsis is more often seen in immunosuppressed patients [45].

The above evidence raises the intriguing possibility that SARS-CoV-2 may be associated with endogenous genetic waste products. By contrast, no causal evidence supports a zoonotic transmission of exogenous viruses in COVID-19 [46]. About half of COVID-19 cases reported diarrhea and digestive symptoms in Wuhan, Hubei Provence, China, where SARS-CoV-2 was first detected in wet markets [47]. Gastrointestinal symptoms are highly unusual and inconsistent with the low amount of these symptoms in patients with severe acute respiratory syndrome (SARS) or Middle East respiratory syndrome (MERS) [48]. Although gastrointestinal symptoms are associated with the consumption of wild animal products [49], there is no causative evidence linking exposure to wild animal products and zoonotic transmission of SARS-CoV-2 from bat SARS-related coronavirus (bat SARSr-CoV) [50]. Of relevance, a study of Dietary Approaches to Stop Hypertension (DASH) found that a high dietary sodium intake was associated with a 27% increased risk of gastrointestinal bloating, compared with a 41% increased risk of bloating associated with a high dietary fiber intake [51]. Bloating from high dietary sodium was independent of dietary fiber intake in the study.

Briefly summarizing the reviewed evidence so far, there are many unknowns in the field of virology that require further investigation. Reviewed evidence in this article supports the mechanisms that excrete and transport endogenous waste products that have the potential to contribute to infectious disease pathophysiology. Of particular interest, viral infections are established by virion aggregates, not single virions, and viral aggregates may form from the undisposed waste products of cells. Furthermore, testing is insufficient to provide proof that the detection of viral RNA in specimens always correlates with viral transmissibility and viral proliferation in ILIs. The remaining sections of this article synthesize the transdisciplinary evidence from virological, immunological, pathophysiological, and epidemiological determinants, linking the molecular mechanisms of sodium toxicity with COVID-19.

## 3. Risk Factors Associated with COVID-19

Most viral infections are associated with other disease-causative agents, in addition to the viral infection itself [52]. Evidence suggests a somewhat independent relationship may exist between a viral infection and disease-causative agents. For example, a weak presence of disease-causative agents could explain why asymptomatic people with detected exposure to SARS-CoV-2 infections do not develop clinically significant disease symptoms. Conversely, a strong presence of disease-causative agents could explain why antibodies from SARS-CoV-2 infection alone may be insufficient to prevent severe disease symptoms in people who are reinfected with SARS-CoV-2 [53].

China and Italy were among the first countries to experience severe outbreaks during the COVID-19 pandemic. Hypertension was identified as a risk factor associated with severe cases of COVID-19 in China and Italy [54]. Of relevance, hypertension was found to be a risk factor associated with mortality in both the 2009 (H1N1) swine influenza pandemic [55] and the 2013 avian influenza A (H7N9) virus infection [56]. Hypertension risk is also associated with a high dietary sodium chloride intake [57], and China is among the highest consumers of sodium chloride in the world [58]. Almost half of the Chinese population between 35–75 years of age has hypertension, most of which is untreated and uncontrolled [59]. Italy also ranks high among consumers of sodium chloride [60], and hypertension prevalence in Italy affects up to 59% of the population over 18 years of age [61].

In addition, Italy has a large ageing population with underlying health conditions, associated with an increased case fatality rate in COVID-19 [62]. Further studies are needed to investigate the population sodium intake associated with severity of COVID-19 outbreaks by country or region. Other large countries with a high sodium intake include India, the United States, Australia, Canada, and England [63].

Cases of COVID-19 in the research literature have been associated with stroke [64], thrombosis [65], cardiac arrhythmias [66,67], obesity [68], diabetes [69], kidney disease [70], hepatic disease [71], multiple sclerosis [72], systemic sclerosis [73], migraine [74], tinnitus [75], Bell’s palsy [76], polycystic ovary syndrome [77], and pneumonia [78]. Incidentally, the coronavirus discovered in Wuhan, China, in December 2019 was first detected in cases of novel coronavirus-infected pneumonia (NCIP) [79]. Likewise, high sodium chloride intake and sodium concentration levels are risk factors associated with stroke [80], thrombosis [81], cardiac arrhythmias [82,83], obesity [84], diabetes [85], kidney disease [86], hepatic disease [87], multiple sclerosis [88], systemic sclerosis [89], migraine [90], tinnitus [91], Bell’s palsy [92], polycystic ovary syndrome [93], and pneumonia [94], providing clinical evidence that these diseases form a transitive link between COVID-19 and a high sodium chloride intake. Transitive inference is a comparative analysis tool described in the present author’s previous work [16]. Inferred transitive links are not strong enough to prove causation, but these links are useful for exploring unknown areas and identifying related subjects for further research. In addition, pediatric Kawasaki-like symptoms associated with COVID-19 have been reported, known as multi-system inflammatory syndrome in children (MIS-C) [95]. As in COVID-19, MIS-C and Kawasaki-like symptoms have also been associated with hypertension [96], stroke [97], acute heart failure [98], and acute kidney injury [99], transitively linking sodium toxicity to pediatric disease pathophysiology. Although other dietary, environmental, and lifestyle factors are relevant to the pathophysiology of COVID-19, numerous transitive links with COVID-19 suggest that sodium toxicity may be among the leading factors potentially contributing to the disease.

Excessive sodium intake leading to sodium toxicity exceeds the human body’s minimum requirement of 500 mg sodium needed to function properly [100], which can be provided from natural foods without added sodium chloride. Sodium chloride contains about 40% sodium by weight, and Americans consume excessive sodium, averaging 3300 mg a day [101]. Although sodium chloride is an ionic compound that dissolves into separate sodium and chloride ions in an aqueous state, electrolysis is required to overcome the electrostatic force connecting sodium and chloride ions in a brine solution, producing poisonous chlorine gas [102]. No evidence exists that electrolysis naturally occurs within the human body to convert sodium chloride to free sodium ions and free chloride ions for physiological functions, such as ion passage through individual ion channels in cell membranes that select only one type of ion [103]. In addition, observational studies claiming increased mortality associated with a low sodium intake were challenged for having research design flaws, such as selection bias from not excluding seriously ill patients, and information bias from poor data collection of the daily sodium intake [104,105]. 

When the body content of water and sodium are excessively high in edematous conditions, hyponatremia (serum sodium levels <135 mEq/L) is caused by dilution from an osmotic shift of water out of cells [106,107,108]. Called hypervolemic hyponatremia, this type of hyponatremia is often treated with diuretics and the restriction of fluids and sodium. Hypervolemic hyponatremia should be differentiated from other types of hyponatremia where the body water content is low, as in hypovolemic hyponatremia, and the water content is normal, as in euvolemic hyponatremia. Disconcertingly, out of a dozen recent articles reporting adverse outcomes and increased mortality in COVID-19 patients with hyponatremia [109,110,111,112,113,114,115,116,117,118,119,120], only two of the articles mentioned hypervolemic hyponatremia [115,119]. Moreover, none of the studies in the 12 articles stratified patients by hyponatremia type. Further research is needed to clarify the prevalence of hypervolemic hyponatremia associated with high water and sodium levels in COVID-19 patients. Of relevance, hyponatremia that occurs in syndrome of inappropriate antidiuretic hormone secretion (SIADH), also reported in COVID-19 patients [115], results from excess water rather than sodium deficiency [121].

Normally, excess sodium is excreted by the kidneys, with additional losses through the skin and gastrointestinal tract, but a high sodium chloride intake may overload renal nephrons with excessive pressure and volume, causing a decline in the glomerular filtration rate [122]. Burdened renal function is associated with impaired sodium excretion, as in chronic kidney disease [123], which may gradually increase the development of sodium toxicity in the body tissues. No specific level of sodium intake has been associated with sodium toxicity. However, according to the U.S. Department of Agriculture and U.S. Department of Health and Human Services’ *Dietary Guidelines for Americans 2020-2025*, the daily sodium chronic disease risk reduction limit for adults is 2300 mg [124], implying that daily sodium intake above this level may increase risk of toxic or pathogenic effects. Lethal dietary sodium levels for some adults (just under four tablespoons of sodium chloride) are almost twice the upper range consumed by some people in China [125]. Although evidence has not previously linked sodium chloride as an associated risk factor for infectious respiratory diseases like COVID-19, a high sodium chloride intake has been associated with severe noninfectious chronic respiratory diseases such as asthma [126] and chronic bronchitis [127]. 

Evidence associating sodium toxicity with thrombosis and stroke in COVID-19 patients is grounded in research findings linking thrombosis to elevated serum sodium in mice, mediated by vascular endothelial cell secretion of the blood-clotting factor, the von Willebrand Factor (vWF) [81]. Researchers have also found that elevated sodium chloride increases vWF secretion in vascular endothelial cell cultures, and analysis of the data from the Atherosclerosis Risk in Communities Study revealed that serum sodium is associated with plasma vWF and stroke risk.

## 4. SARS-CoV-2 Infection and Sodium Toxicity

The cells of the respiratory system are susceptible to infection through exposure to viruses and other pathogens; however, a non-specific innate immune response and an adaptive immune response eliminates viruses and protects respiratory cells from infection [128]. As part of the mucosal immune system, the nasal mucosa provides physical protection against dehydration and injury from mechanical and chemical agents, and provides the clearance of particles and microorganisms [129]. 

However, sodium toxicity adversely affects the nasal mucosal immune system, which may lead to respiratory viral infection. For example, hypertonic concentrations of 2% sodium chloride were used in vitro to experimentally induce ciliostasis, paralyzing cilia beating in the nasal mucosa epithelial cells, which normally transport the mucous out of the airways. When the epithelial cells were infected with influenza A virus, the viral yield in the saline-treated cells increased two- to three-fold compared with the untreated cells, demonstrating that normally beating cilia impede viral infection [130]. Although 2% NaCl in these experiments is 2-fold and 20-fold higher than normal extracellular and intracellular concentrations, respectively, findings may be translated bench-to-bedside for more moderate clinical applications. Specifically, moderately impaired function of the cilia caused by sodium toxicity could inhibit mucociliary clearance, leading to increased accumulation of viruses in patients’ nasal passages, as detected in laboratory analyses of nasopharyngeal swab specimens collected during COVID-19 testing [131]. COVID-19 patients were found to have prolonged mucociliary clearance compared with healthy ear, nose, and throat outpatients with non-nasal symptoms [132]. Furthermore, the upper nasal passages are a potential portal allowing viruses and other particles to enter the bloodstream, eventually leading to viral sepsis [44]. Of relevance, other research has found that an increased concentration of plasma sodium in the hypernatremia was significantly associated with sepsis in elderly patients [133]. This evidence could explain sepsis in COVID-19 patients associated with sodium toxicity [44].

No difference in viral load was found in asymptomatic infections of SARS-CoV-2 and infections with symptoms in Lombardy, Italy [134], and no viral load differences were found across patient gender, age, and disease severity in Guangzhou, China [135]. This evidence suggests laboratory confirmed viral infections of SARS-CoV-2 and clinical symptoms of COVID-19 may have separate causative pathways, which would explain why they do not always appear together. For example, asymptomatic infections occur without clinical symptoms [136], and clinical symptoms in post-acute COVID-19 syndrome persist after the acute infection stage subsides [137]. These facts support a hypothesis proposing that the association of COVID-19 and SARS-CoV-2 is mediated by a related disease determinant. On the other hand, compared with mild cases, the mean viral loads were 60 times higher in the nasal passages of cases associated with the most severe symptoms in Nanchang, China [138], which could be related to severely impaired viral clearance in the nasal mucosal immune system due to excessively strong sodium toxicity. Of relevance, the viral clearance was delayed and clinical outcomes did not improve when corticosteroids were used to treat SARS-1 patients [139], and corticosteroids cause retention of sodium [140]. Likewise, the French Health Ministry suggested that ibuprofen aggravates infection in COVID-19 [139], and non-steroid anti-inflammatory drugs (NSAIDs) like ibuprofen cause sodium and water retention [141].

## 5. Sodium Toxicity and Immune Response in COVID-19

Sodium chloride intake is associated with changes in immune responses that promote organ damage and inflammation, including increased release of inflammatory cytokines, like interleukin (IL)-6, macrophage inflammatory protein-2 (MIP-2), and tumor necrosis factor (TNF)-α [142]. Elevated sodium chloride concentrations also increase the proliferation of T-cells, while decreasing the anti-inflammatory responses—for example, anti-inflammatory M2 macrophages are suppressed while pro-inflammatory M1 macrophages are increased by high sodium chloride levels. Furthermore, sodium chloride was found to enhance the production of IL-4 and IL-13, and to suppress the production of interferon-γ (OFN-γ) in memory T cells [143]. Interleukin-17 (IL-17)-producing helper T cells (Th17) play a role in clearing the extracellular pathogens, and Th17 cell development is induced by a kinase signaling pathway activated by high sodium chloride concentrations [144]. 

Researchers have reported that a “high-salt diet promotes skin Na+ accumulation, which boosts macrophage activation”, leading researchers to “speculate” that cutaneous sodium storage provides a barrier against infection [145]. However, researchers have also noted “that skin Na+ deposition is linked with disease in humans”. Alternatively, macrophage activation suggests an inflammatory immune response to salt-induced tissue damage, as “high-salt diets result in interstitial hypertonic Na+ accumulation in the skin and muscle that activates tissue-resident macrophages” [146]. 

The secretion of cytokines in response to RNA viruses, like SARS-CoV-2, include TNF-α and IL-6, with a general imbalance toward pro-inflammatory responses in contrast with antiviral responses [147], similar to the responses to sodium toxicity. Resilient T cell immunity is necessary for the efficient control of viruses, and T cell counts in COVID-19 patients with mild symptoms were found to be normal or a bit higher—again, an immune response similar to sodium toxicity. However, T cell counts were reduced in moderate and severe cases, suggesting that the T cell response is dysregulated in severe cases, possibly because of exhaustion from over-activation. This reduction in T cell counts could be related to severe sodium toxicity, and more investigations are needed in this area. Of relevance, human receptor angiotensin-converting enzyme 2 (ACE2) is a binding site for SARS-CoV-2, and ACE2 is found in the membranes of alveolar macrophage cells in the respiratory tract and in other cells throughout the immune system [148], potentially providing a protective mechanism that facilitates endocytosis and the lysis of pathogens. However, this protection could be compromised, as ACE2 expression is reduced under conditions of high sodium chloride dietary intake, as was found to occur in the renal system in animal experiments [149]. In addition, based on the studies of other human coronaviruses, humoral responses to coronavirus infection are comparatively short-lived and provide only partial protection from reinfection. Considering the failure to find a cure for coronavirus infections in the common cold after many decades of research [150], the potential for newly developed vaccines to eliminate the virus and its variants does not portend well [151].

## 6. Sodium Toxicity and COVID-19 Symptoms

**Figure 1:**
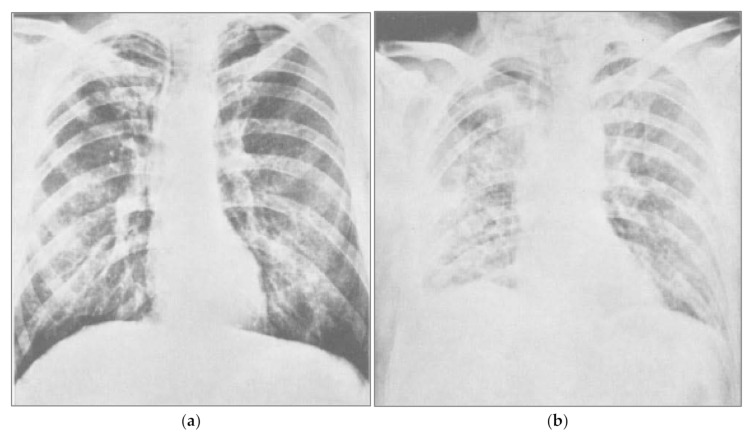
Pulmonary congestion following sodium chloride infusion. (**a**) Patient before a large infusion of sodium chloride and water in a 1969 controlled clinical trial. (**b**) Same patient two days after infusion, showing severe congestion from pulmonary edema due to fluid retention. Reprinted from Hutchin et al., 1969 (132). Pulmonary congestion following infusion of large fluid loads in thoracic surgery patients, The Annals of Thoracic Surgery, 8(4), 339–347, with permission from Elsevier.

COVID-19 symptoms include fever, dry cough, fatigue, headache, and nasal congestion, and more serious symptoms include difficulty breathing [152]. Regardless of whether symptoms are due to acute cases or long-haul COVID-19 [153], sodium toxicity may play direct and indirect roles in many of these symptoms. In 1969, a controlled clinical trial tested the hypothesis that large infusions of sodium chloride and water would prevent fluid loss and hypovolemia related to complications in thoracic surgery [154]. After receiving sodium chloride infusions, some patients rapidly developed severe pulmonary congestion and fluid retention in the lungs, or pulmonary edema, which blocked respiration and lowered arterial oxygen pressure (pO_2_; Figure 1). One patient in the study died, and other patients who received infusions containing various concentrations of sodium chloride were placed on ventilators. These clinical experimental results demonstrate the harm of excessive sodium chloride and retained fluid in the lungs, causing pulmonary congestion, hypervolemia, and edema. The chest X-ray taken after sodium chloride infusion (b) is similar to the radiographs of drownings [155]. 

Ground-glass opacities are often observed in the lungs of COVID-19 patients [156], and further research is needed to investigate whether related fluid in the lungs may increase to harmful levels in COVID-19 patients if sodium is retained under conditions of sodium toxicity. A recent review of fluid therapy in thoracic surgery confirmed that aggressive fluid infusion promotes hypervolemia, interstitial edema, and impaired oxygen diffusion [157], and the aggressive accumulation of fluid is associated with mortality and acute kidney injury in pneumonia from influenza A H1N1 virus [158]. The clearance of alveolar edema fluid requires active transport of sodium out of the airspaces, with water removal following the osmotic concentration gradient [159]. Intraperitoneal injection of distilled water (1500 mL) and glucose have been reported to help in the recovery of acute salt poisoning cases [160], probably by reducing the hypertonicity of the extracellular fluid caused by concentrated levels of sodium.

Clinical evidence from a retrospective cohort study in Wuhan, China, shows that acute respiratory distress syndrome (ARDS) is a risk factor for mortality associated with SARS-CoV-2 [161]. ARDS shares symptoms of severe shortness of breath with COVID-19, yet the causes listed for ARDS do not include viral infections [162]. Italian physicians noted that ARDS is not typical in COVID-19 patients compared with other ARDS patients [163], and ventilation may do more harm than good. For example, in Fluid Management in the Ventilated Patient, Bersen wrote,

“…various neurohumoral responses to positive-pressure ventilation lead to retention of sodium and water, as a homeostatic response to raised intrathoracic pressure. A major consequence of this response is expanded plasma volume, and a tendency toward systemic and pulmonary edema [164].”

The air sacs of the lungs in fatal cases of COVID-19 with ARDS were reported to become filled with a gummy yellow fluid that blocked the transfer of oxygen to the blood from the lungs, even though a ventilator was pumping in oxygen [165]. Under these conditions, adjusting ventilation to force in higher amounts of oxygen under greater pressure can damage the lungs. In-hospital mortality in critically ill COVID-19 patients increased with high frequency use of ventilators in two major hospitals in New York City [166]. Non-invasive ventilation procedures that cause less lung damage have since gained “a more significant and positive role” in treating COVID-19 patients [167]. Furthermore, the gummy yellow fluid in the lungs of COVID-19 patients appears identical to the yellow fluid identified in pulmonary edema [168]. Pleural effusion is also a pulmonary condition observed in Kawasaki Disease [169]. Although other factors like toxins and medications are linked to pulmonary edema [170], the above findings strengthen the link between pulmonary edema and COVID-19. Furthermore, because sodium toxicity is also linked to pulmonary edema, a potential transitive link is established between sodium toxicity and ARDS in COVID-19 patients.

In view of the potential harm from pulmonary edema that may be related to sodium toxicity, adverse effects from administering intravenous saline in patients with ILIs like COVID-19 warrant further investigation. Warnings from the U.S. Food and Drug Administration for 0.9% sodium chloride parenteral intravenous injection include “risk of solute overload causing congested states with peripheral and pulmonary edema” [171]. Serious adverse effects of normal saline solution also include shortness of breath, joint pain, rash, fever, and tachycardia [172]. Favorable outcomes that reduced death and renal dysfunction were found when switching intravenous fluids from saline to balanced crystalloid fluids, especially in cases of sepsis [173]. Additionally, almost a quarter of COVID-19 patients have acute heart failure, half of whom have no history of hypertension or cardiovascular disease, and aggressive fluid treatment is not recommended in these cases [174]. Furthermore, earlier research reported that patients having a type of acute heart failure with pulmonary edema had severe dyspnea, tachycardia, alveolar edema, and reduced arterial oxygen saturation, and adverse side effects of diuretic therapy in these cases were related to poorer outcomes [175].

Other ILI symptoms associated with sodium toxicity in COVID-19 are related to hypervolemia, or fluid overload, a condition of excess sodium and water in the blood, which expands the extracellular fluid compartment in the body [176]. Hypervolemia most commonly manifests as edema, and sodium restriction is essential for avoiding this condition. The World Health Organization reported that blocked sinuses in acute sinusitis fill with fluid and can cause headaches [177]. The edema of the nasal mucosal is a clinical feature of acute rhinosinusitis or inflammation of the nasal sinus mucosa, which is claimed to be caused by viruses [178]. The association of viral infections with rhinosinusitis may be mediated by sodium-induced hypervolemia and nasal mucosal edema, which could explain why nasal congestion and headache is associated with SARS-CoV-2 infection under conditions of sodium toxicity [90,179,180,181,182].

Migraine is associated with COVID-19 [74]. Researchers have demonstrated increased sodium permeability through the blood−brain barrier and blood cerebral spinal fluid barrier during migraine [90], and future research should investigate an association between this mechanism and sodium dietary intake. Dysregulated voltage-gated sodium channels are proposed to increase neurovascular compression on the roots of trigeminal cranial nerve fibers and cause migraine pain [179], and an association between this dysregulated mechanism with dietary sodium intake should also be investigated. On the other hand, an inverse association was found between dietary sodium intake and migraine [180,181], although researchers cautioned against treating migraine patients with sodium [182]. Further research is needed to more fully investigate the relationships between dysregulated sodium, dietary sodium intake, and migraine.

Loss of the sense of taste, ageusia, and smell, anosmia, also occurs in COVID-19 [183], which may result from blocked sensory olfactory receptors in congested nasal tissue related to sodium toxicity. Edematous congestion may also contribute to peripheral nerve compression and entrapment, causing nerve damage [184], as in Bell’s palsy induced by compression from saline injection [92].

**Figure 2:**
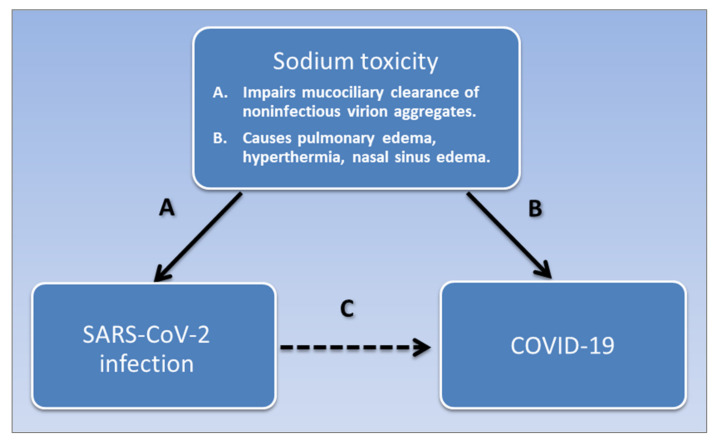
Sodium toxicity potentially mediates the association of SARS-CoV-2 infection and COVID-19. SARS-CoV-2 is associated with COVID-19, shown in pathway **C**. Sodium toxicity is a common causative factor that impairs the mucociliary clearance of noninfectious virion aggregates in the nasal mucosa, causing SARS-Cov-2 infection, shown in pathway **A**. Sodium toxicity also causes pulmonary edema, hyperthermia, and nasal sinus edema in COVID-19, shown in pathway **B**. Pathway **A** is potentially weak or absent in post-acute COVID-19 syndrome, and pathway **B** is potentially weak or absent in asymptomatic infections.

Furthermore, animal studies have shown that injected sodium chloride acts as a pyrogen that causes fever, which researchers have suggested was induced by an imbalance between sodium and calcium ions in the anterior hypothalamus that controls hyperthermia [185]. As well as acting as an emetic that induces vomiting [186], adverse effects of pharmaceutical sodium chloride tablets include fever and rashes [187]. Skin rashes are dermatologic manifestations of COVID-19 [188], and higher salt concentrations were found in the skin of people with atopic dermatitis [143]. Thus, sodium toxicity accounts for many of the symptoms of ILIs like COVID-19. Figure 2 summarizes how sodium toxicity potentially mediates the association of SARS-CoV-2 and COVID-19.

Of relevance, normal saline (0.9%) was used in placebo injections during randomized controlled trials for mRNA COVID-19 vaccines [189]. Just as very small amounts of certain foods, drugs, and environmental antigens can trigger hypersensitivity reactions in some people [190], small 0.3 mL saline injections could trigger mild hypersensitivity in apparently healthy people with underlying sodium toxicity. With over 15,000 to 20,000 placebo participants in the Moderna and Pfzier/BioNTech trials, respectively [191,192], a very small percentage of participants affected with mild hypersensitivity to saline, having adverse effects similar to COVID-19 symptoms [172], could potentially confound the trial results. Furthermore, the mRNA vaccines contain polyethylene glycol (PEG) [189], which has immunosuppressive and anti-inflammatory effects [193] that could suppress mild hypersensitivity reactions to saline used to reconstitute the Pfizer vaccine [194]. These issues should be explored further.

## 7. Determinants of Sodium Toxicity

Seasonal ILI epidemics annually claim hundreds of thousands of lives across the globe [195]. Seasonal changes in sodium balance, related to sodium dietary intake and excretion, are associated with sodium toxicity, which may help explain seasonal incidence of influenza and ILIs such as COVID-19. A recent study of seasonal influenza mortality found significant inverse associations with temperature, sunlight, and precipitation, supporting influenza patterns during late winter and early spring in temperate climates, and influenza patterns during the rainy season in subtropical and tropical climates [196]. Seasonal changes in behavior have also been proposed to account for non-environmental effects on seasonal influenza [197]. For example, research in Israel found that workers generally gained bodyweight and consumed over 40% more sodium chloride in the winter compared with summer [198]. Sodium intake also increased in winter in older adults in Turkey [199], in adults in Southern Brazil [200], and in a middle-aged and elderly Dutch cohort [201]. In Japanese hypertensive outpatients, 24-h urinary sodium chloride excretion, a reliable biomarker of sodium intake, generally decreased in the summer [202]. Other seasonal nutritional factors related to increased food intake, including seasonal changes in body mass index and waist circumference [203], may increase vulnerability to illness, such as the excessive consumption of dietary fat and carbohydrates for energy and heat maintenance during colder seasons. Of relevance, grains, baked goods, and meats, which are relatively rich in carbohydrates and/or fats, contribute the majority of sodium chloride to the American diet [204].

Climate is also associated with the prevalence of COVID-19; for each one-degree increase in latitude from the equator, the associated prevalence of COVID-19 cases increases by 4.3% per one-million inhabitants [205]. Increasing latitude is also associated with multiple sclerosis (MS) in U.S. women [206] and, as previously mentioned, MS is associated with both COVID-19 and dietary sodium [72,88]. Although a prospective study found no association between sodium intake and MS [207], the study did not compare a high-sodium diet with a low-sodium diet containing <1500 mg sodium, as recommended by U.S. health authorities [208]. Furthermore, populations in East Asia, South East Asia, and South Asia have been reported to have lower MS incidence and prevalence, despite a high sodium intake; however, flaws and weaknesses in the diagnosis and data collection methods in these areas encumber accurate MS surveillance [209].

Males working in moderately hot conditions for an average of 10 h lost 4.8 to 6 g of sodium through sweat [210]. People eating a high-salt diet excrete more sodium in sweat than people eating a low-salt diet [211]. Near-daily aerobic activity that increases heart rate, breathing, and sweating for at least 20 min, such as playing basketball, is correlated with reduced frequency, severity, and symptomology of upper respiratory tract infections [212]. Of relevance, no professional basketball players tested positive for COVID-19 during the first five weeks of the 2020 National Basketball Association playoffs during the pandemic [213], which may be related to the athletes’ profuse sweating of up to more than four liters during each game [214]. In addition, Artic warming is linked to the increased frequency of extreme winter conditions in the Eastern United States [215], which could intensify or extend behavioral patterns of higher sodium chloride intake during cold weather, thus contributing to the frequency of severe ILIs associated with sodium toxicity.

Salt, fat, and sugar added to highly processed foods also contribute unhealthy commodities that increase the risks associated with non-communicable disease pandemics affecting low-income and middle-income countries [216,217]. However, the potential of sodium chloride in processed foods to cause pandemics of infectious diseases is under-investigated. Even felines in zoos are fed processed canned foods containing added sodium chloride [218], and SARS-CoV-2 infection has been detected within these animals [219]. 

During World War I, before modern food preservatives or commercial frozen foods were available, sodium chloride was the preservative of choice in canned foods, which were just coming onto the market [220]. Canned foods replaced fresh foods that were rationed during the war and were no longer available to the general public [221]. The 1918 influenza pandemic, which killed an estimated 20–50 million people, began in March as a typical seasonal influenza and weakened over the summer. A second wave returned in the fall when it increased in virulence, with reports describing “blistering fevers” and how “patients would drown in their own fluid-filled lungs” [222]. With so many deaths, “many questioned whether such an explosively fatal disease could be influenza at all” [223]. “War edema” during WWI was linked to European populations consuming “large amounts of fluid and salt in the attempt to sustain life on the thin vegetable soups common in prison camps and in famine districts” [224].

Doctors from the U.S. Navy in Boston, Massachusetts, in 1918 exposed 62 healthy male sailors to a variety of direct contact with the sputum, coughs, and exhaled breath from severely ill influenza patients [225]. Although one healthy sailor developed a sore throat, none of the healthy sailors became ill with confirmed cases of influenza. A similar experiment was later repeated in San Francisco, California, and the results were negative, providing strong evidence that the influenza virus was noninfectious. More recent literature reviews found scant or no evidence of influenza or SARS-CoV-2 transmission in controlled experiments involving asymptomatic and presymptomatic patients [226,227], providing further supporting evidence that RNA viruses in ILIs are noninfectious. These clinical findings challenge conventional views claiming that influenza infections spread through droplets containing individual viruses [228]. Furthermore, “increasing evidence indicates that viruses do not simply propagate as independent virions among cells, organs, and host” [229], and individual virions rarely establish infections [42]. Rather, large aggregates of virions within the body are required to overcome immune barriers and mediate viral infection [37].

After World War I ended and fresh foods were no longer rationed, the influenza pandemic eventually subsided in 1919. Of note, placing patients directly in the warm sun all day was part of an effective open-air treatment for influenza during the 1918 pandemic [230], which is consistent with previously cited sodium losses through the skin in moderately hot conditions. Furthermore, an early controlled trial found that sauna bathing decreased the frequency of the common cold [231], and saunas were also associated with decreased incidence of hypertension in a prospective cohort study in Finland [232], which could be related to sodium losses through sweating.

Sea voyages extending back many centuries were notorious for high mortality rates among ship crew and passengers, and onboard food provisions were often highly salted [233]. “Mariners recognized a connection between their diets and their health”, and fresh fruits and vegetables were acknowledged to cure “scorbutic disorders which are contracted by salt diet and long voyages” [234]. In 1915, many crew members of the German ship SS *Kronprinz Wilhelm* suffered debilitating malnutrition, with edema, shortness of breath, and pneumonia, on a diet high in salted meat and processed foods [235]. Even when carrying more fresh fruit and vegetables, modern cruise ships appear susceptible to infectious disease outbreaks, evidenced by the coronavirus outbreak aboard the cruise ship *Diamond Princess* [236]. The procurement of food on board liner ships should avoid over reliance on processed foods in response to supply chain challenges of food degradation and “shelf life constraints for raw materials and perishable products” [237]. 

Today’s high cost and restricted availability of fresh unprocessed nutrient-rich foods like whole fruits and vegetables, compared with less expensive and highly-salted processed foods, could account for increased COVID-19 infections within lower socioeconomic communities [238]. A recent systematic review and meta-analysis of studies investigating healthy adult populations found that people of lower socioeconomic status consume 14% more sodium than people of a higher socioeconomic status [239]. Southwestern American Indians in particular had the highest mortality rates of all racial-ethnic groups in the 1918 influenza pandemic and the 2009 H1N1 pandemic, and have had exceedingly high hospitalization rates, younger patients, and disease severity in the COVID-19 pandemic [240]. The Southwestern American Indian population also has disproportionally high rates of mortality from diabetes, obesity, and cardiovascular disease, and the population’s “inadequate access to healthy foods” [240] suggests a potential link to sodium toxicity. 

Furthermore, seasonal influenza outbreaks in long-term care facilities are “well recognized” [241], as occurred during the COVID-19 pandemic [242]. Nursing homes have been associated with inconsistent climate control conditions [243], and COVID-19 outbreaks in prisons are attributed to overcrowding [244]. Inferior institutional food service is also common in government-run nursing homes and prisons [245].

“Our taxpayer-funded, government-run institutions focus on meeting legal nutritional guidelines for the lowest price… Many people facing illness or incarceration are without options or a voice. They are reduced to eating the most processed, least nutritious food available, entirely because it’s cheapest for the rest of us” [245].

Even people living at home who avoid public markets during a pandemic may face nutritional challenges from a “greater consumption of processed, nonperishable foods which can be high in sodium” [4]. These findings imply a need for government subsidies to lower consumer prices of fresh, unprocessed whole foods, and increase the availability of these foods to vulnerable populations. Along with nutritional education, such a national nutritional policy would greatly benefit public health and help prevent non-communicable diseases, as well as potentially prevent infectious diseases throughout the nation. Furthermore, interventions to reduce COVID-19 morbidity and mortality through reduced-sodium diets should be explored, particularly targeting long-term care homes with vulnerable populations.

## 8. Conclusions

This perspective article used a grounded theory method to synthesize evidence from virology, immunology, pathophysiology, and epidemiology literature, inferring that sodium toxicity is a modifiable factor potentially associated with COVID-19 morbidity and mortality. A high sodium chloride intake is a risk factor associated with diseases that are also risk factors of underlying conditions associated with COVID-19 morbidity and mortality. The most fatal pathological effect of COVID-19 is acute respiratory distress syndrome with yellow fluid that blocks lung air sacs, and this appears to be the same fluid in pulmonary edema caused by excess sodium chloride. Sodium toxicity is also associated with other symptoms of COVID-19 like fever and nasal sinus congestion. Virion aggregates form from undisposed cellular waste, and most virion aggregates in influenza A contain noninfectious viruses. Similar aggregates of SARS-CoV-2 may accumulate in the upper nasal passages due to impaired mucociliary clearance from the ciliostasis-effect of sodium chloride. Sodium toxicity in highly processed foods also potentially contributes to infectious influenza-like illnesses, and further exploration of low-sodium diets to reduce COVID-19 morbidity and mortality are needed.

The limitations of this paper include the nascent literature on COVID-19, which, although plentiful, has not been sufficiently investigated to generate great depth, maturity, and diversity of knowledge. In addition, as with any theory, the concepts described in this paper are subject to verification through hypothesis testing, and the need to modify theoretical concepts is likely to occur as more evidence accumulates. The transdisciplinary nature of the paper also draws upon a broad range of knowledge domains, and is not intended as a substitute for more advanced papers in these areas. Finally, although the grounded theory method attempts to minimize subjectivity, each researcher has a unique point of view, and similar papers on this topic by other authors are needed to contribute diverse perspectives.

## Data Availability

Not applicable.
